# Is green economy achievable through championing green growth? A local government experience from Zambia

**DOI:** 10.4102/jamba.v8i3.253

**Published:** 2016-03-11

**Authors:** Phiri Rodgers

**Affiliations:** 1 Department of Language and Social Sciences, University of Zambia, Zambia; 2 Zambia Network for Environmental Educators and Practitioners, Lusaka, Zambia; 3 Department of Language and Social Sciences, University of Zambia Environmental Education Students, Zambia

## Abstract

The need to enhance environmental sustainability, sustainable development and growth that takes into account the well-being of the people and nature because of the increased production and consumption of goods and services is the major driver to the introduction of green economy in Zambia and countries in southern Africa. This article examines the extent to which local government in Zambia has embraced green growth and green economy and critically analyses the concept of green economy and green growth. This study is based on a review of planning and policy documents, a household questionnaire survey and interviews with various institutions, planners and rural development organisations. A number of policies implemented at the local government level were analysed and reflected upon irrespective of whether they contain the components of green growth and green economy and the extent to which they contribute to attaining green economy. The article argues that the need for economic diversification is important as far as green economy is concerned. The article recommends the need to invest in research and development in order to find more carbon-free economic activities. The conclusion is that local government is key to achieving green growth and green economy, because it is involved at all levels, from policy formulation to implementation.

## Introduction

Green growth implies nurturing economic growth and development at the same time, ensuring that natural assets such as terrestrial, aquatic and atmospheric ecosystems continue to provide the resources and environmental services on which our well-being depends (Organisation for Economic Co-operation and Development – OECD 2011:5). To do this, it must catalyse investment and innovation which will underpin sustained growth and give rise to new economic opportunities. Green Growth is believed to be the supplement, or rather the requirement and ingredient, for green economy and sustainable development. It is argued that green economy cannot be achieved without championing green growth first (OECD 2011:8). Green growth is therefore considered as the path and need to achieve sustainable development and green economy.

Green economy and green growth are topics of the day currently. The ‘green economy’ concept has gained priority in various intergovernmental forums such as the United Nations Environment Programme’s (UNEP) Green Economy Initiative, the OECD Green Growth Strategy and in discussions amongst G20 leaders. In addition, green economy in the context of sustainable development and poverty eradication has been declared a priority theme for the United Nations Conference on Sustainable Development in 2012 (Rio+20; UNCSD) (Bass 2013:11). Several intergovernmental organisations are making it a priority and are announcing policies and programmes, but they each have different approaches and priorities.

However, the Zambian definition for green growth is development that makes sustainable and equitable use of Zambia’s natural resources within ecological limits through reinforcing the three cornerstones of sustainable development, specifically which are the economy, social welfare and the environment, basically the development processes that do not surpass the resources that the earth can provide (United Nations Conference on Sustainable Development – UNCSD 2012:46). Despite Zambia having this definition of green growth, Chigunta and Matshalaga (2011:6) stated that there are still some irregularities in the system of production of goods and services, which will later be discussed within the article. For instance, in Zambia carbon emission and deforestation have received very little attention by the local government, amongst other issues.

Local government is a system of public administration which in a mainstream of perspectives exists as the lowest layer of management within a given state (Ismail, Bayat & Meyer 1997:2–3). Local government may also be defined as a public organisation authorised to decide and administer a limited range of public policies within a relatively small territory, which is a subdivision of a regional or national government. Local government is at the bottom of a pyramid of governmental institutions, with the national government at the top and intermediate governments (states, regions and provinces) occupying the middle range. Local governments generally act within powers delegated to them by legislation or directives of the higher level of government.

In the case of Zambia, central government is represented throughout Zambia by the provincial government system, each of whom is the president’s direct representative and appointed by the president to each of the provinces. The 10 provinces are divided into districts, each of which has a district council (DC) chairman responsible to the provincial deputy minister. The DC chairman is particularly concerned with political and economic developments. His (DC chairman) civil service counterpart is the district executive secretary. The cities of Lusaka, Ndola and Kitwe have councils and mayors, but the formerly separate management of mine townships on the Copperbelt has been abolished (Chikula & Sichone 1996:17).

In the actual sense, it is the local government which is responsible for ensuring that policies implemented are sustainable and do not contribute to the rise in carbon emission and overutilisation of environmental resources, because they are the direct link between the central government and the status of the environment as a result of the policies implemented. In most instances, African countries formulate green policies, but the problem comes in during the implementation process and mostly it is the local government performing this function (Chigunta & Matshalaga 2011:14).

This article comes in eight major sections including the introduction, conclusion and recommendations. The next section is dedicated to addressing methodological foundations. Section two focuses on literature review of green economy and green growth in Zambia, as well as an analysis of why green growth is key to achieving a green economy. The results from the self-administered questionnaires, interviews and legislative frameworks are outlined under section three. The results are discussed under section four. Section five outlines information on the economic situation in Zambia. Section six is the conclusion. It revisits the main points from the article. The last section outlines the recommendations. It outlines the suggestions from the key informants on how Zambia can achieve a green economy.

## Research methodology

This article aims to determine how and to what extent local governments in Zambia are engaging with the green growth agenda as envisioned by the national (and provincial) government. It also aims to identify and discuss the factors that determine or affect local government engagement with this agenda, its adoption by local government and its contribution to the socio-economic well-being of Zambia’s population. The information collected within reflects the extent to which the local government engages with green growth and the factors that affect its engagement with this agenda.

Both primary and secondary data were used. Primary data were acquired using self-administered questionnaires amongst counsellors, village headmen, youths, women and leaders of cooperatives. Eighty-five questionnaires in total were administered to these respondents. This research methodology aimed to collect information on the extent to which local government has implemented green economic policies in the surrounding communities.

Secondary data were collected from books, journals, districts and village organisations and private companies that are involved in the production of goods and services. The purpose of this research method was to find out the exact meaning of green growth and green economy.

Secondary data also include the information collected through the survey of legislative framework. Information was collected on the nature of policies at the local government level and their implementation and effectiveness in relation to green economy. The policies to be implemented at the local government level include provision of clean water to all members of the communities. Information was also collected to determine whether there are any constraints such as lack of funding for green policies and poor infrastructure to achieve green growth.

Apart from the methods mentioned above, interviews were also used and key informants include extension workers, staff of district planning officers and government officials involved in monitoring and managing the environment and production processes taking place in the environment (if they degrade the environment or cause increase in carbon concentration in the atmosphere). The interviews also aimed to find out if there any solutions to deforestation that the government has embarked on. Precision information about water quality, green jobs creation, as well as the deforestation caused by current massive construction of infrastructure was obtained from the field. Interviews aimed to collect information on the extent to which the local government know and embrace green economy.

Other sources of information used in this article include books and direct consultation from University of Zambia environmental education students and lecturers.

### Literature review

The idea of green economy is basically an emerging paradigm, especially amongst developing countries, since its inception by United Nations Environment Programme (UNEP 2012:15). However, it is slowly becoming an integral part of the developmental plans and processes amongst countries. This paradigm of green economy has some close characteristics with sustainable development. According to Karpagam (2004:4), sustainable development is defined as the improvement in the quality of life that meets the present needs without compromising the ability of future generations to meet their needs. The question which arises now are what then are green economy and green growth?

Bass (2013:23) argued that green economy refers to an economic system whose economic activities result in value-added human welfare and social equity, at the same time focusing on reducing environmental risks (environmental risks such as increase in carbon emission and degradation of the environment) and ecological scarcities (low supply of resources by the environment because of overexploitation of its resources). It is that economy which does not put the lives of the people in danger. Because of its economic activity, it ensures a balance amongst the ecological, political and economic aspects of the environment through reducing the negative impact of one sector upon another. Bass (2013:8) stated that ‘green economy has a number of objectives of which some of them were stated out from the definition’. The major objective is sustainable and efficient use of environmental resources without exceeding the sustained yield; for instance, the need for an improved human welfare, equity, sustainable resource management, resilience and ability to adapt to climate change. Green economy is also based on the principle of qualitative growth, the type of growth which does not compromise the society, for instance, the system of production processes comprising low carbon and environmentally friendly technologies. Green economy is also considered as that economy whose increase in income and employment perpetuated by the private and public sectors results in a reduction in carbon emission and pollution levels.

The green economy framework calls for a strengthened relationship amongst the three pillars of sustainable development, which are economic sustainability, environmental sustainability and sociopolitical sustainability (International Chamber of Commerce 2011:3). This implies that whilst economic activities (activities that involve the production, distribution and consumption of goods and services) are taking place in an economy, there is supposed to be a sustainable growth, sustainable utilisation of environmental resources and sustainability on the sociopolitical aspect as well, implying that decisions made should contribute to environmental sustainability and reduction in carbon emission. These three pillars of sustainable development are interlinked and therefore cannot function without the other. However, Green Economy calls for consideration of each of the three pillars of sustainable development so that economic activities do not affect the environmental and social aspects negatively, and vice versa, thereby contributing to self-regeneration of the environment. It has also been realised that green economy cannot be achieved without following a given process of production. It is for this reason that green growth was proposed as a process through which green economy can be made possible. An economy that is characterised by green processes and practices will result in green growth, it will also be realised that green economy is achieved. It operates in such a way that it removes pressure from the environment and alleviates it to commodity prices.

According to UNCSD (2012:46), green growth is a terminology used to refer to the increase in the production of goods and services accompanied with a reduction in carbon emission and sustainable utilisation of natural resources. It focuses on structuring the economy in a way that it combines economic growth and environmental protection, thereby building a green economy in which investments in resource savings as well as sustainable management of natural capital are drivers of growth. An economy which is in closer alignment with sustainable development objectives provides opportunities for using financial resources better to meet development needs and reducing the vulnerability of socio-economic systems to environmental change and resource constraints.

#### Why green growth is key to achieve a green economy

Low (2011:1) views green growth from the sight of stronger sustainability. In stronger sustainability, the environment is at the centre of everything whilst the social, economic, technological and governance pillars come after the environment and all need to speak to sustaining the environment as a life support system. The environment and the economy are not supposed to be viewed as mutually exclusive and conflicting spaces. This implies that the social, economic, technological and political pillars are supposed to increase the production of goods and services without degrading the environment. It is realised that in order to achieve green growth, there is need to lower greenhouse gas (GHG) emission and put in place climate change adaptation measures as a way of tackling climate change (Nhamo & Nhamo 2014:58). The purpose of ensuring lower GHG emission as an economy in achieving economic growth is to bring up spaces for low carbon growth. Green growth also calls for interaction amongst economic growth, human development and poverty reduction, which will result in equitable growth. Human development, poverty reduction, enhanced biodiversity and ecosystem services lead to the strengthening of communities and habitats, which is the core of green growth.

The targets of green growth described above are essential for the achievement of a green economy. This is because when the targets of green growth are achieved, the results will improve human well-being and social equity through human development and poverty reduction, whilst significantly reducing environmental risks through lower GHG and climate change adaptation measures. Ecological scarcities will also be reduced through enhanced biodiversity and ecosystems services. From this, it be stated that green growth is important for the achievement of a green economy. Thus, green growth as a process and green economy as an end result are not questionable as it is evident from the above paragraph and cannot be further interrogated.

The linkage between green growth and green economy and climate change is very strong. This is because the factors that are eliminated in the production system to achieve green growth and green economy are the same factors that cause climate change. For instance, green economy emphasises the use of clean technology (carbon-free technology), on the other hand – one of the ways of preventing climate change is to avoid releasing carbon dioxide (avoid burning of waste which can decompose) which the atmosphere cannot absorb. Green growth also calls for the use of green practices and being sustainable, such as using renewable energy. Climate change adaptation also calls for consideration of adaptation measures in communities such as sustainable consumption of water and use of renewable energy such as solar energy.

Green economy in Zambia is a movement that is supported by almost all government departments and ministries and works hand in hand with a number of countries in the region and world over. Despite the efforts made by Zambia in embracing green economy, the nature of companies – especially Foreign Direct Investment (FDI) – do not operate in ways that ensure reduction in carbon emission or put in place measures such as carbon sequestration, thereby hindering green growth. Agencies such as the Zambia Environmental Management Agency (ZEMA) do not carry out their roles and duties based on merit because they are being influenced by individuals having political power (The Post 2014). For instance, when they reject a proposal which tends to be detrimental to the environment, decisions from people with political powers tend to overrule the decision by ZEMA. Mostly, local government is involved in choosing sites for establishing companies such as manufacturing and mining companies. Agencies such as ZEMA are there to carry out environment impact assessment, and when the positive impacts outweigh the negative impacts, the proposed project is supposed to proceed.

The concept of green growth offers competitive advantages to those countries that commit to policy innovations. For instance, Brand Green Zambia, which is a clear programme of continuous improvement in policy across sectors towards green and inclusive standards, attracts quality investors and buyers who favour sustainable and ethically produced products, which are increasing (Banda & Bass 2014:24–25). A green brand in one sector can help and is helped by green images in others, an integrated approach that will support more sustainable consumption and production. Priority themes include instilling a water-wise culture in population through water harvesting and children’s active involvement in initiatives such as waste-to-power and improving environment inclusion in the curriculum. It also pushes for best standards for imported mining equipment and less carbon emission machines such as cars. There is everything to be gained by using the international standards (*International* Organisation for Standardisation) that manufacturers have to follow in most markets both for imports and exports. The aim should be to shift from dirty private transport to cleaner and more efficient public means, for instance, electric rail investment, top quality buses and bicycles.

The Permanent Secretary for the Ministry of Finance, Mr. Felix Nkulukusa, indicated that there:

is need for Zambia to define what green Economy or growth is, having struggled as a nation with the divergent international approaches at Rio+20. The minister offered the idea that green growth could combine two priorities: generating inclusive growth and tackling climate change. The Permanent Secretary stressed that there is need to identify those green approaches that support Zambia’s strategic priorities, notably aspirations of inclusive growth. He pointed out that energy and agriculture have been identified as critical sectors for inclusion, already have green dimensions, but need bankable green options to be elaborated, while institutional collaboration around climate change needs to be firmed up as well, quoted by. (UNCSD 2012:9)

In championing green growth, Zambia through the ‘The Zambia Green Jobs Programme’ has focused on Green building, also known as green construction or sustainable building. International Labour Organisation (ILO 2012:7) stated that green building refers to the construction and use of processes that are environmentally responsible and resource-efficient throughout a building process, from the stage of siting to designing, construction, operation, maintenance, renovation and demolition. It involves the construction of buildings sustainably such that they are durable, comfortable and usable. It also involves using materials that are renewable, rather than nonrenewable, thereby reducing pressure on the nonrenewable resources and encouraging conservation of natural resources.

Another Southern Africa Developing Country to be cited in this article is the Republic of Zimbabwe. Through the Medium Term Plan for the period 2011–2015, the Government of Zimbabwe is also working to change the economy, create jobs, maintain macroeconomic stability and restore the capacities to produce goods and services competitively. The government has also aimed to ensure environmental sustainability and social benefits for all (United Nations 2012:2). The Zimbabwean government has realised that the need to build a green economy is critical for the country’s ability to provide a sustainable means of livelihood to its population who live in rural areas and derive their livelihoods from potential sectors such as agriculture. Zimbabwean government also aims to increase access to sustainable energy as it is estimated that only 37% of households in the country have access to reliable energy. The Zimbabwean government has also realised the need to generate decent and green jobs to challenge high rates of unemployment predominantly amongst the youth who represent over 50% of the population. The United Nations in Zimbabwe is in total support of these national efforts through the Zimbabwe United Nations Development Assistance Framework for 2012–2015.

## Results

### Contribution of Zambia’s major economic activity to green economy

The research was conducted within Zambia. This region of Africa is concentrated with a number of economic activities ranging from food, building materials, domestic products, hospitality, health, tourism, mining and agriculture. These sectors face a number of difficulties to be managed sustainably. Evidence is based on a number of cases such as that where investors were given a license to establish a mine in the source of the Zambezi River, despite some protests from environmental organisations, including the independent environmental governing body ZEMA, the mining company was granted a license by the courts (The Post 2014). Zambia’s central mining area, Copperbelt province from time immemorial, is still recording some cases of pollution by the mining companies. The mining companies have claimed to have implemented environmentally friendly technologies to reduce the rate of air and land pollution. The number of mining companies is increasing but the amount of money spent on improving the environment is very little and not creating employment in the area. As such, the health of the people in this area is at risk because of excessive pollution. The income and employment created from the mining sector are therefore not sustainable and not a characteristic of a green economy.

### Data collected by questionnaires

The Zambian government has created a number of jobs in the construction sector; most youths are employed under the road construction projects which are not long term and it is these same projects which have resulted in thousands of trees to be cut to pave way for a road, of which tree planting is not part of the objectives.

**FIGURE 1:**
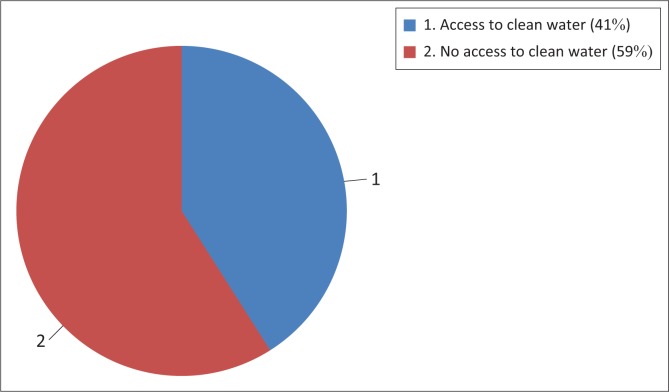
Percentage of people accessing clean water in Zambia.

**FIGURE 2:**
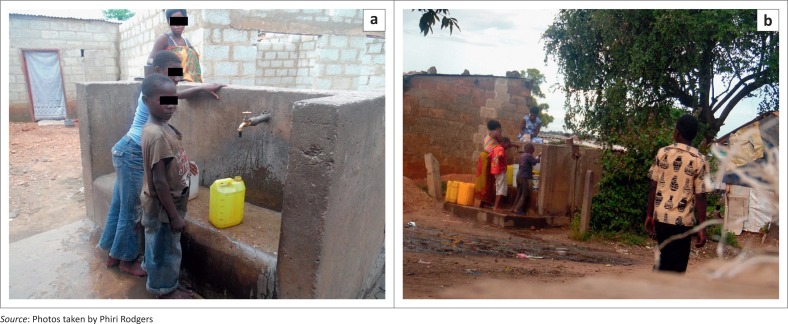
Water crisis (a and b) in garden compound.

Access to clean water, however, has also been seen as a challenge since only 41% of the population of Zambia has access to clean drinking water (Figure 1). As illustrated in Figure 2, children still walk two miles and make queues to access clean water in rural areas and most unplanned settlements such as Kanyama, Misisi, Garden and George compounds face water challenges. The progress in access to clean water is very slow. The resources generated by the Zambian government to enhance national development and their outcomes are used to improve the lives of the people, which is the goal and objective of green growth.

**FIGURE 3:**
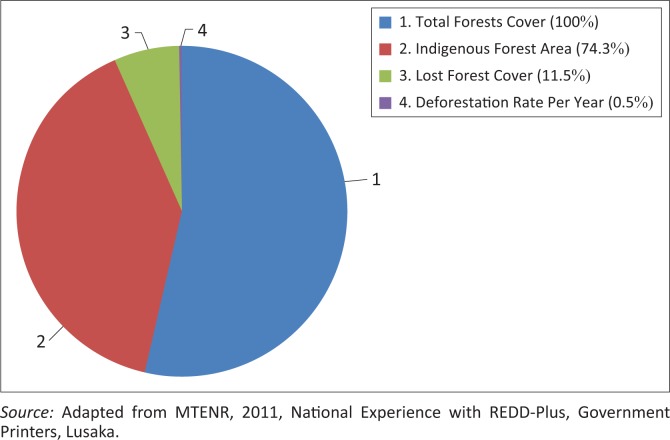
Deforestation rate in Zambia.

**FIGURE 4:**
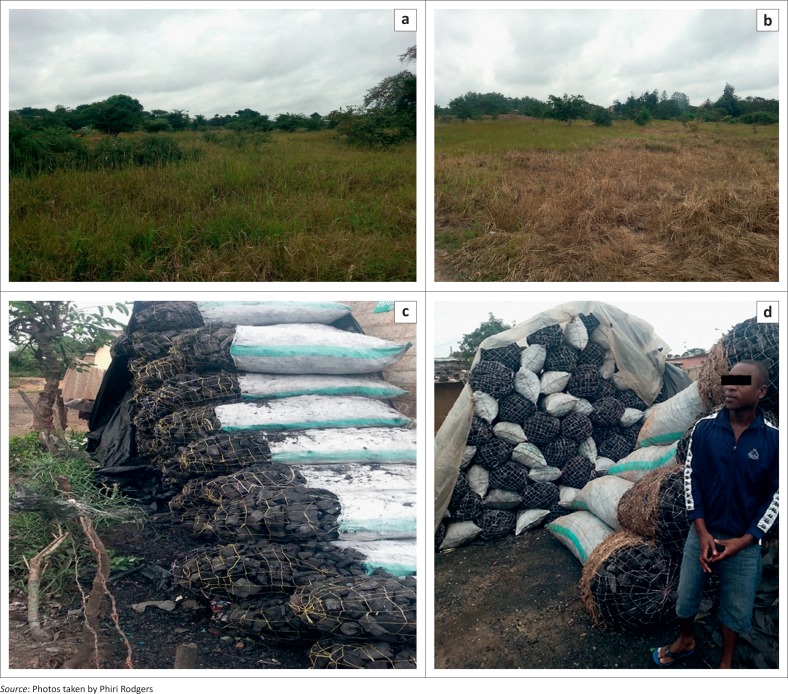
Deforestation and charcoal burning in Zambia: (a) showing the boundary of trees 6 months before, (b) showing how the boundary of trees has moved further after 6 months, (c) showing charcoal made from logs and (d) charcoal made from trees being cut from the forests.

A total of 45.8 million ha of land has been affected by deforestation at a rate of approximately 300 000 ha per year, as illustrated in Figure 3. Zambia, in 2012, banned the trading of timber after the country realised that the trading was not sustainable. The ban is still in effect up to now, although some people are reported to trade and burn charcoal illegally. In the early 2014, the director of forest department took the task in hands to inspect and arrest the illegal charcoal burners in Chongwe district of Lusaka Province. The effects of cutting trees for charcoal on forests are as shown in Figure 4 (Ministry of Tourism Environment and Natural Resources – MTENR 2011:30). Trees are not given enough time to grow. As a result, only shrubs are remaining in both natural and man-made forests.

### Data collected by interviews

Zambia has chosen the sustainable path and has shown commitment despite budget constraints and poor infrastructure. Government leadership has already removed unsustainable fuel subsidies in order to reallocate resources to health and education. Zambia now needs to find the small, quick wins as well as the big leaps towards green growth whilst creating green jobs and products and taking them to the markets with the right technologies.

For the pilot activities in the field of sustainable agriculture, Zambia promoted agro-forestry. It had set a goal of increasing the number of commercial farmers employing sustainable farming practices by 20% and, for small- to medium-scale farmers, by 30% in the assessment of waste, collection systems, transportation, final treatment methods and facilities. It also provided a basis for mainstreaming sustainable consumption and production in sectoral policies and strategies. An example of the sustainable practices they promoted is tree intercropping. One of the most effective species for intercropping in Zambia is *Faidherbia albida* (locally known as Musangu) (Food and Agriculture Organization 2011:14). This tree has been frequently reported to significantly increase crop yields for cotton, groundnut, maize, millet and sorghum when grown in proximity. The African Carbon Credit exchange worked with Zambian and international partners to get each farmer to agree to plant 100 *Faidherbia albida* on one ha of their land.

To promote efficient use of energy the government, in cooperation with Zambia Electricity Supply Corporation Limited, started an electricity demand-side programme. Some of its actions include the suspension of duty and value-added tax on energy-saving lamps, energy-efficient appliances and power generators. The government also introduced a voluntary ‘Time of Use’ tariff for high-demand users, such as farmers and manufacturers. These users receive a discount of between 25% and 50% on capacity and energy charges, respectively, between 10 p.m. and 6 a.m. This measure is believed to free up capacity for power during the day. Gauri and Mohamed (2012:2) cites this as one of the key components of green economy, which is the reduction in the use of hydroelectricity and use of renewable energy sources such as solar.

Zambia Rural Electrification Authority has intensified the supply of electricity in rural areas. Through this programme, a number of rural people have been given access to electricity. The installation of electricity in rural areas is being coupled with the installation of solar geyser so as to reduce the demand of hydroelectric power and make use of the vast sunlight in the country (MTENR 2011:29). One major constraint is poverty, which is also still rampant in Zambia, limiting the majority of its population from access to clean renewable energy, because of the lack of affordability. The off-grid market can benefit the majority if it is driven by nonmainstream investors and is often supported by short-term donor support, which means that projects rarely survive after donor support ends.

### Legislative framework

Zambia through the local government has the overarching framework for sustainable development which is ‘Vision 2030’. Its principal polices that integrate sustainable consumption and production elements are the National Policy on Agriculture (2005), the National Policy on Agriculture (2007), the National Energy Policy (2008) and the National Water Policy (2010) (MTENR 2011:17–20). One key objective of the Zambia Vision 2030 is to establish a fully integrated and sustainable water and resource management programme.

In efforts to improve the water sector, Zambia aimed to improve access to appropriate and environmentally friendly sanitation for all, using the Devolution Trust Fund to fund pilot projects in low-income areas. The Devolution Trust Fund was established in 2003 by the National Water Supply and Sanitation Council and is funded by the Government of the Republic of Zambia. It funded water kiosks, which were set up by commercial utilities to provide basic sanitation. In 2010, it provided the funding to set up 65 water kiosks, 59 km of water pipes and water tanks with 350 m^3^ capacity; one kiosk can serve up to 1800 people (MTENR 2011:46).

Zambia has made considerable progress towards the integration of economic, social and environmental analysis in the planning process as evidenced by the National Development Plans (NDPs). The two most recent plans are particularly cases in point, that is the Fifth (2006–2010) and Sixth (2011–2015) NDPs. These Plans have key sectors and subsectors in the country that constitute the economic, social and environmental pillars. For instance, amongst others, the economic pillar in the two National Development Programmes is represented by mining, construction, commerce trade and industry, transport and manufacturing. The focus of the social pillar includes social protection, health, education, and child and youth development. On the other hand, the environmental pillar targets sectors and subsectors that include the following: agriculture, livestock and fisheries, natural resources, tourism, water and sanitation and energy (NPE 2012:20–25).

## Discussion of the results

Achieving green growth and green economy depends on how the local government prioritises these concepts, high prioritisation of green growth by local government entails successful path to green economy. This is because high prioritisation of green economy results in maximum effort in the implementation of green policies and putting in necessary measures for the achievement of green growth, thereby leading to green economy. Apart from creating employment through the mining sector, Zambia is also creating jobs through the construction, tourism and hospitality sectors. Zambia has implemented a number of projects in which it has managed to employ many youths. These jobs are short term and so cannot be said to be sustainable or green jobs because they do not fully contribute to human development and poverty reduction. This makes it difficult to achieve green growth because the environment is degraded because of high pollution levels such as burning of charcoal.

This sector has also contributed to the loss of biodiversity in Zambia and restocking of species is not mostly considered and the loss of biodiversity such as trees implies the loss of carbon sink. For instance, the construction of shopping malls, roads and estates has contributed much to the loss of trees and animal species. This is an indication that to some extent, Zambia does not value natural capital. As much as this may be considered growth, the money used to implement these projects is borrowed from World Bank and International Monitory Fund, making Zambia one of the Highly Indebted Poor Countries. The study reviews that in order for Zambia to pay back its debt, it is degrading its environment. Degrading the environment to achieve green growth is not possible because the utilisation and allocation of resources is not sustainable.

Even though there are great economic activities in Zambia, the companies operating within the communities’ payback less in improving the well-being of the people. The resources which the companies use are obtained from the local community. It is their mandate to ensure that the communities are benefiting by helping them solve the problems faced such as lack of health services, clean atmosphere (carbon free) and providing resources for youth skill training such as making monuments’ using litter. This can also be done by increasing access to clean water because majority of the rural people do not have access to clean drinking water. Local government has worked with a number of private firms to provide water in rural areas through establishing communal taps. The companies (especially mines) pollute the rivers (Kafue River) and air in the surrounding communities (Kankoyo community) by increasing the amount of carbon compounds in the air and rivers. This cannot be said to be green growth because it does not contribute to the well-being of the people. It puts their health at risk and ignores the targets of green growth, which are low carbon growth and equitable growth. It is also not in line with the information outlined in the literature review that green economy emphasises practices that benefit the three pillars of sustainable development.

Zambia is embracing green economy by reducing dependence on charcoal as a source of energy, which has contributed to a loss of forests. Through the local government, Zambia is providing electricity to people in rural areas as well as installing solar geysers to encourage the use of solar energy. Local government has also started installing solar street lights to make use of the vast sunlight the country has. Zambia’s effort to achieve green economy has been observed through its emphasis on the use of carbon-free sources of energy. This move by Zambia of trying to reduce dependence on charcoal which causes deforestation is in line with the target of green economy which is tackling climate change. Deforestation is an agent of climate change because it destroys natural carbon sinkers. Initiating of these projects by the Zambian local government indicates the important role that local government can play in achieving green economy.

Results from the legislation indicate that the nature of polices Zambia is formulating are green. This can be seen from the policies such as the National Energy Policy (NEP 2008:23). Zambia is being helped by the increasing number of environmental organisations. These organisations advise the government in as far as green growth is concerned. Green economy policies can be formulated, but poorly implemented if the local government does not embrace green economy. This is the problem facing Zambia because it has many green economic policies, but they are poorly implemented. The weakness of the local government in Zambia can be said to have contributed in Zambia’s delay to achieve green economy.

The results clearly indicate that there is lack of diversification in the Zambian economy and this makes it difficult to achieve green growth because there is a tendency of developing one sector at the expense of the other. This situation is made worse by lack of consistency in the implementation of policies by the local government.

### Economic situation in Zambia

The research was conducted in Zambia, starting from the local government level where the implementation is supposed to be more effective and influential, to the top officials carrying out the planning and budgeting. Zambia has tried to embrace green growth and green economy through the nature of policies they are implementing and putting in place at the grass-root level. As Zambia strives hard to meet the objectives of green economy, there are constraints, failures and successes. However, green growth is turning out to be a prioritised paradigm in Zambia’s policy consideration.

Green economy is a movement which is supported by almost all government departments and ministries, and through working hand in hand with a number of countries in the region and world over. Despite the efforts made by Zambia in embracing green economy, the nature of companies especially FDI do not operate in an environmentally friendly means, thereby hindering green growth (Centre for Trade Policy and Development – CTPD 2012:1). For instance, in Zambia, it has been observed that the focus of government has mainly been on attracting FDI through lower tax rates and an assortment of incentives, experience has shown that the presence of the mining industry in an area often breeds underdevelopment, land displacement, violation of human rights on the people employed in mines, poverty, environmental degradation, health, and other social problems (CTPD 2012:5).

Agencies such as the ZEMA do not perform their roles and duties without restrictions because they are being influenced by political power, such as when they reject a proposal which tends to be detrimental to the environment, political influence tends to overrule. As such, there is need for the local government in Zambia to be made aware of the objectives and requirements of green economy (CTPD 2012:10). The local government is very much important because it is supposed to notify the central government about the state of the environment and the extent of integration amongst the three environmental pillars.

The amount of money which is allocated towards the goals of green growth is insufficient and makes these agencies such as ZEMA not to achieve their objectives. There is also poor funding of institutions, For instance, government is supporting the Farmer Input Support Programme which is inorganic fertiliser based, thereby undermining the long-term soil fertility level. At the same time, Government is supporting Conservation Agriculture which promotes use of organic fertiliser amongst others. As such, there is conflict on the nature of policies implemented by the local government (UNCSD 2012:53). On the other hand, political power seems to be prioritised in Zambia thereby suppressing the demands of green growth. The research shows that there is no proper coordination between the central government and the local government in Zambia.

The supply of electricity in rural areas which is being combined with the installation of solar geysers is an effort to reduce the rate of deforestation in Zambia. Despite this move, the tree planting policies are not fully supported and lack consistency at local government level so as to replace the lost trees, because they are done on commemoration days only. Zambia Electricity Supply Corporation (ZESCO) is also intensifying efforts to make use of the abundant solar energy in the country by identifying other demand areas such as street lights and traffic lights (Zambia Electricity Supply Corporation 2011:5). This is indeed one of the objectives of green economy to make proper use of renewable energy and the modular nature of solar energy technologies make them particularly suitable for capital-constrained countries like Zambia. However, it would be beneficial for Zambia to initiate a dialogue that would include all relevant stakeholders in the development of an integrated resource plan for power generation that will help guide the country in diversifying its electricity mix whilst meeting future demand. A premise to such an activity is the undertaking of a thorough renewable energy resource assessment.

Poor implementation of environmental policies is another weakness to achieving green economy, the document *Environmental Management Act* of 2011 (EMA 2011:122–150) outlines the rules and regulations on how activities in the environment are to be undertaken. It is the duty of the local government to ensure that firms operating locally are carrying out their functions according to the regulation and guidelines outlined in the *Environmental Management Act* of 2011. The local government has to be consistent in monitoring the operations of the companies in order to ensure that environmental rules and regulations are being followed and implemented. The research also indicates that there is lack of ability to follow the development path taken by those countries that have developed whilst protecting their environment.

Embarking on providing or creating green jobs would help Zambia meet the objectives of a green economy. Green Jobs can be defined as work in agricultural, manufacturing, research and development, administrative and service activities that contribute significantly to preserving or restoring environmental quality. Precisely, this includes jobs that help to protect ecosystems and biodiversity; reduce energy, materials and water consumption through high-efficiency strategies; decarbonise the economy; and minimise or altogether avoid generation of all forms of waste and pollution (ILO 2012:7).

## Recommendation

The awarding of contracts and licenses should be given to those local and foreign corporations which are willing to operate in an environmentally friendly way, unlike the current situation where profit is the major goal.

The environment should be prioritised and not politicised, because many mining firms in Zambia are given licenses even when ZEMA rejects the proposal after carrying out an Environmental Impact Assessment.

There is need to stimulate research and development in Renewable Energy Technologies by government creating a Research and Development fund for Renewable Energy Technologies to be used by the University of Zambia Energy Research Centre and the National Council for Scientific Research. The research should be centred on development of appropriate and affordable Renewable Energy Technologies for rural areas. In addition, collaboration between researchers, policy makers and planners should be strengthened for the common purpose of promoting renewable energy technology. This will reduce deforestation and pressure on hydroelectric power.

There is need for the government to take advantage of Environmental Educators in the country, in sensitising environmental sustainability and educating the communities about environmental issues which are becoming rampant such as poor waste management, high pollution levels and lack of environmentally friendly habits and attitude starting from individual level to national level, as well as encouraging the use of solar equipment to make use of the abundant sunlight that we have in Zambia.

Zambia should enhance its preparedness to access and organise funds from local and international sources including the Green agenda for improved mobilisation and sequencing of finances for economic and environmental management. The domestic and international financing should be used to catalyse and leverage private sector investment in addressing the challenges to achieving a green economy. Zambia has a wide range of renewable energy sources (solar, hydropower, biomass, wind, geothermal and energy crops) with great potential.

Tree planting should be embraced by the government by ensuring that more trees are being planted than the number which is being harvested, basically employing the sustained yield principles, not harvesting more than 50 % of the resources. Planting of trees should be made a daily activity by ensuring that everyone celebrating his or her birthday to plant the number of trees according to his or her number of years on that day, starting from the President, cabinet officers, members of the government and all the citizens of Zambia.

## Conclusion

It is clear that Zambia has enormous renewable energy resources that can play a significant role in meeting the energy needs of the country. There is need to revisit the plans and policies by making them more sustainable and green growth oriented. Considerable efforts have been made towards the implementation of sustainable development in the country as evidenced amongst others by the establishment of the ministry of environment, NDPs and other institutions aimed at promoting sustainable development following the Rio 1992 recommendations and to some extent incorporate sustainable development pillars (economic, social and environment). There is also sustainable development debate in general that is beginning to be expressed through such issues as diversification from mining to other sectors with regards to economic development. Efforts are being made towards the promotion of Conservation Agriculture and public concerns on pollution particularly by the mining houses. Zambia’s struggles in achieving green economy can be seen from the efforts stated above. The country’s development agenda emphasises social innovation using national development strategies that are multifaced in terms of their incorporation of economic, social and environmental issues. The most significant of these strategies are the NDPs and the Vision 2030, which are important and contribute to achieving green economy. In order to end deforestation especially in rural areas, Zambia implemented the rural electrification programme and the installation of solar geysers to reduce pressure on the hydroelectric power. The country is facing a number of challenges in achieving green economy, which require support from the local government which plays a major role in policy implementation. It can be concluded that strengthening the local government in Zambia is key to achieving green growth and green economy.
